# 3D Imaging Advancements and New Technologies in Clinical and Scientific Dental and Orthodontic Fields

**DOI:** 10.3390/jcm11082200

**Published:** 2022-04-14

**Authors:** Rosalia Maria Leonardi

**Affiliations:** Department of Medical-Surgical Specialties—Section of Orthodontics, School of Dentistry, University of Catania, Policlinico Universitario “G. Rodolico- San Marco”, Via Santa Sofia 78, 95123 Catania, Italy; rleonard@unict.it

The recent technological updates in medical field are irrevocably changing the clinical work-flow of dentists, from the diagnosis to the treatment plan approaches and decision-making activities. New available technologies permit the accomplishment of a comprehensive multidisciplinary approach to the rehabilitative treatments as well as enhance the effectiveness and efficiency of the therapy and streamline daily clinical workflow.

3D Imaging systems represent one of the pillars of this new era for dentistry and dental applications. In this regard, Cone-Beam Computed Tomography (CBCT) has represented a significant step forward in the diagnostic imaging protocols. 3D digital reproduction of maxillofacial anatomy from CBCT are used in the diagnosis and planning of therapy [1], surgery [2], dental implantology [3], and in the cranio-facial research field [4,5,6,7,8,9,10,11,12]. In addition, intra-oral scans and facial scans allow for reproduction, respectively, the dentoalveolar component and facial soft-tissue and to integrate these data with those obtained from CBCT, for a comprehensive “digitally-assisted” diagnosis and treatment plan [13,14,15,16].

The step further the usage of digital reconstruction of the anatomical structures is the possibility to edit and modify these files, following appropriate biological and anatomical principles and landmarks/references, in order to acquire useful information for the analysis of the morphology and for comparative pre- and post- treatment evaluation. This can be achieved by using software, borrowed from informatics engineering and based on sophisticated algorithms, that can perform the superimposition [17,18] of the anatomical digital files and the calculation of the surface distances. Such differences are visualized in a color-coded map which allows clinicians to distinguish area of disagreement between the two surfaces, representing the morphological differences or the changes induced by a specific therapy. Data differences can also be expressed in percentage of agreement between the two 3D rendered models. This technique is called deviation analysis and surface-to-surface matching [19,20].

Artificial Intelligence (AI) has been introduced with the aim to enhance diagnosis, therapy, prognosis, and patient monitoring [21]. Artificial intelligence (AI) is the capacity of a computer to accomplish tasks normally associated to humans. Hence, AI itself is a general term that defines the imitation of human intelligence. Computer vision is one of the most dominant types of AI which is already involved in digital technology used during our daily-life, sometimes without being aware of it [22]. Computer vision is based on replicating parts of the human visual system, allowing computers to identify and process entities in images and videos. Categorization, definition of areas, entity detection and identification represent the typical computer vision tasks of this system. The usage of computer vision is becoming widespread among clinicians to enhance diagnosis, monitoring the progression/regression of diseases, and set the appropriate treatment protocols [23]. To date, the most prevailing applications for computer vision in medical field are those related to radiology and imaging systems [24,25].

The latest innovations in 3D imaging and rapid prototyping (RP) procedures are significantly modifying the clinical workflow of dental and orthodontic specialists [26]. With the appropriate equipment, it is possible to improve the efficiency and the accuracy of dental manufactures production [27,28] such as dental models for fabricating dental aligners [29,30,31,32], occlusal and splints [33,34], bonding trays [35,36], positional guide for miniscrew insertion [37,38], on lays or veneers [15], etcetera. 3D printing is a manufacturing process involving stratification of materials to produce an object. It can be based on different material processing technologies, with the Vat-Polymerization system being the most popular for orthodontic and dental applications [39,40,41]. In the Vat-Polymerization system, the liquid resin is polymerized with exposure to a light source [6], and according to the light source employed, 3D printers are classified as stereolithography (SLA), digital light processing (DLP), and liquid crystal display based (LCD).

There are many options for dental 3D printers including cost-effective desktop models and industrial high throughput machines. The main advantages of 3D printing applications in dentistry are:

(1) Better Fit to The Patient: 3D printers can create intricate structures and allow for greater geometric complexity without sacrificing production time. As 3D printed oral devices are more customizable, they are more accurate and better fit the patient without requiring extensive trimming and polishing.
(2) Simplified Production Processes: Dentistry has long embraced digital manufacturing technologies to simplify production processes and workflows. Early developments with intraoral scanners allowed dentists to make and send oral impressions to a dental lab within minutes. Today’s 3D printers eliminate thermoforming. Clear aligners, retainers, nightguards, and other devices are directly 3D printed with minimal post-production. Dentists and lab technicians save time, labor, and material by eliminating models, thermoforming, and product trimming.
(3) In-Office Printing: Dentists that integrate the 3D printing machines into their operations gain better control over workflow and minimize product turnaround time. Creating oral devices in-house saves money on lab fees and shipping costs while also enabling same-day patient services for specific devices.

Virtual reality is a interesting tool which to date has been under-utilized in dentistry. VR can be described as “a computer generated, three-dimensional world in which the user interacts with virtual objects” or characters [42,43], permitting the full combination of user’ cognitive, motor and mental functions. Virtual reality technology can be generally categorized into immersive virtual reality and non-immersive virtual reality. Both these two categories of VR can be applied in dentistry field, with immersive virtual reality being used as a distraction tool for patients during procedures, while non-immersive virtual reality being used for surgical or clinical procedure simulations [44].

Concerning patients’ experience, VR supports the children’s’ confidence and reiterates their ability to face complex or challenging situations. This would lead subjects experience to the next dental visit with less anxiety.

Concerning doctors’ experience, the aim of VR is to provide a consistent and secure platform for the analysis of different anatomical areas for diagnosis, planning and for the surgical training [45]. In this regard, Augmented Reality is the new frontier of “near reality” that integrate virtual reality with a 3D real environment specific for each patient. This occurs through sophisticated registration process which augments the virtual scene with the reality. The integrated image is superimposed on the real environment using semi-transparent glass [46]. This system is particularly interesting for surgical training since the 3D reconstruction of the several tissues provides a realistic platform to work with.

Pain management and patients’ clinical experience represents two other pillars of the new era of dentistry. In this regard, low-level laser therapy is a treatment with no adverse effects that has many functions in medicine and dental practice [47]. It is based on a low-powered laser light source within the red to near-infrared range (wavelengths from 632 to 1064 nm) to induce biological reactions. LLLT has been found to effective in favoring pain reduction after wound healing and during orthodontic treatment [45,47,48,49].

Another approach for pain management is Kinesio Taping (KT). This technique was conceived by Kenzo Kase in Japan in 2003 [50], and it is based on the administration of Kinesio Tapes—thin, waterproof, adhesive, elastic tapes. The use of KT provokes the activation of mechanoreceptors, by pressuring and stretching the skin, and enhances blood and lymph circulation by pulling the subcutaneous tissue and skin from the muscles [50,51,52,53,54,55,56,57,58]. The effectiveness of KT remarkably reduces after 24 h. Kinesio Taping (KT) was found to moderate the gravity of complications after third molar extractions. Recent evidences would suggest that this method could reduce the pain after tooth extraction and trismus and could reduce the dimension of facial edema.

In general, technologies applied to dentistry are in a continuous updating process. Specific editorial initiatives, aiming to collect the best scientific evidence on this topic, are warmly encouraged to allow readers/clinicians to keep up with significant advancements in this topic.
